# Variation in Female Grey Seal (*Halichoerus grypus*) Reproductive Performance Correlates to Proactive-Reactive Behavioural Types

**DOI:** 10.1371/journal.pone.0049598

**Published:** 2012-11-16

**Authors:** Sean D. Twiss, Charlotte Cairns, Ross M. Culloch, Shane A. Richards, Patrick P. Pomeroy

**Affiliations:** 1 School of Biological and Biomedical Sciences, Durham University, Durham, United Kingdom; 2 Sea Mammal Research Unit, Scottish Oceans Institute, University of St Andrews, St Andrews, Fife, United Kingdom; University of Plymouth, United Kingdom

## Abstract

Consistent individual differences (CIDs) in behaviour, indicative of behavioural types or personalities, have been shown in taxa ranging from Cnidaria to Mammalia. However, despite numerous theoretical explanations there remains limited empirical evidence for selective mechanisms that maintain such variation within natural populations. We examined behavioural types and fitness proxies in wild female grey seals at the North Rona breeding colony. Experiments in 2009 and 2010 employed a remotely-controlled vehicle to deliver a novel auditory stimulus to females to elicit changes in pup-checking behaviour. Mothers tested twice during lactation exhibited highly repeatable individual pup-checking rates within and across breeding seasons. Observations of undisturbed mothers (i.e. experiencing no disturbance from conspecifics or experimental test) also revealed CIDs in pup-checking behaviour. However, there was no correlation between an individuals’ pup-checking rate during undisturbed observations with the rate in response to the auditory test, indicating plasticity across situations. The extent to which individuals changed rates of pup-checking from undisturbed to disturbed conditions revealed a continuum of behavioural types from proactive females, who maintained a similar rate throughout, to reactive females, who increased pup-checking markedly in response to the test. Variation in maternal expenditure (daily mass loss rate) was greater among more reactive mothers than proactive mothers. Consequently pups of more reactive mothers had more varied growth rates centred around the long-term population mean. These patterns could not be accounted for by other measured covariates as behavioural type was unrelated to a mother’s prior experience, degree of inter-annual site fidelity, physical characteristics of their pupping habitat, pup sex or pup activity. These findings are consistent with the hypothesis that variation in behavioural types is maintained by spatial and temporal environmental variation combined with limits to phenotype-environment matching.

## Introduction

Consistent individual differences (CIDs) in behaviour, indicative of individual personalities [1]–[6], are now evident in a remarkable array of non-human taxa, from Cnidaria [7] to Mammalia [8], [9]. This apparent ubiquity of personality across a wide spectrum of the animal kingdom indicates that personality is a fundamental evolutionary condition under strong or persistent selective pressure [1]–[3], [5], [6], [10], [11] or is a product of constraints on plasticity that are widespread and, therefore, fundamental for our comprehension of evolution [12], [13]. However, there remains little consensus on the mechanisms underlying CIDs, with debates over whether personalities result from mechanistic constraints [12], [13] or reflect individually differing adaptive solutions to complex physical and social environments [1]–[3], [10], [11], [14]–[16]. An important question is how CIDs in behaviour are maintained in the face of selection [1], [16], [17], and a plethora of theoretical adaptive solutions have been postulated [16], including, frequency and/or state dependent mechanisms [1], life history trade-offs [14], [18], [19], spatial environmental variation combined with limits to phenotype-environment matching [16], bet-hedging in temporally variable environments [20] and non-equilibrium dynamics [16], [21]. Empirical studies of CIDs and their consequences have focused largely on captive individuals, with relatively few investigations having been performed *in situ* [7]. The extent to which behavioural types (defined as the particular behavioural configuration of an individual [3], or their behavioural profile, *sensu* Groothius and Trillmich [22]) expressed in captivity reflect actual behavioural patterns in the wild remains unclear [23], [24], [25], [26]. Links between CIDs in behaviour in the wild and captivity may well be species specific [27]. Therefore, there is a requirement to understand how CIDs in behaviour interact with environmental factors to determine individual fitness in natural populations [2], [3], [5], [7], [9]–[11], [28]–[30].

Grey seals (*Halichoerus grypus*) are one of the few species of marine mammals in which CIDs in behaviour have been shown in free-living wild populations [9], [31]. Grey seals are polygynous, colonial and annual breeders with a discrete, predictable reproductive season. In the UK, adults aggregate to breed each autumn typically at remote island sites [32], where individual females birth and nurse their single pup. Female dispersion patterns on the colony are determined by their pupping site preferences for fine-scale habitat features, in particular access to small pools of water necessary for behavioural thermoregulation [33]–[35]. Males provide no parental care, but compete to maintain home ranges among female aggregations in order to gain copulations when females enter oestrus towards the end of lactation [36]. Grey seals in the UK are capital breeders, relying on stored reserves (mainly blubber) accrued prior to the breeding season to sustain activities on the breeding colony, most importantly for mothers, lactation [37]. In general, mothers with larger post-partum mass can expend more resources on their pup, and given that there is no difference in lactation duration, they achieve higher pup growth rates [37]. In addition to nutritional provisioning, mothers also provide their pups with protection and social interaction [38]. Previous studies of breeding grey seals at the island of North Rona (59° 06' N, 05° 50' W), Scotland, have used observational approaches to show CIDs in male alert behaviour (when a male has his head raised, and is looking around, often in response to some threat or disturbance on the colony) [9] and in-field experimental tests to demonstrate CIDs in pup-checking behaviour of mothers (when a mother is alert, with her head off the ground and makes a definite directed look at her pup) [31].

Here, we determine whether CIDs in pup-checking behaviour persist over consecutive breeding seasons and how individuals modulate their pup-checking behaviour across an undisturbed and a disturbed situation. We use the term situation as defined by Sih et al. [2] to describe differing levels of disturbance within the broader context of parental care. However, it should be noted that there are different definitions in the literature of what constitutes a situation or a context. Some portray contexts as functionally differing behavioural categories such as feeding or parental care whilst the term situation is used to refer to differing environmental conditions within contexts [2], [39]. Others consider context and situation as synonymous (i.e. referring to “all of the external stimuli surrounding an individual when it expresses a given behaviour” [40], [41]). Likewise, there are differences over what constitutes personality. Consistency in behaviour over time has been accepted as adequate evidence [1], [2], [7], whilst others argue that consistency should be expressed both over time and contexts [40], [41]. The two are inevitably linked as an individual can only be in one context (or situation) at any one time. Therefore, we prefer to use the term CIDs in behaviour as this carries fewer connotations, and merely describes observed patterns in behavioural data. Conceptually, this reflects Groothius and Trillmich’s [22] contention that the behavioural patterns observed are the outward manifestations of underlying neurobiological characteristics (which arguably provide a more appropriate basis for classification of personalities).

Consistency in behaviour also implies a limit to plasticity in behaviour [12], [13], therefore, we also examine how individuals differ in their degree of consistency across the undisturbed and disturbed situations, and use this to define behavioural types that can be described along a proactive-reactive continuum. The proactive-reactive axis has been demonstrated in many studies, though almost all are laboratory based [26], [42]. In general, proactive individuals tend to be more aggressive, form routines more readily and express relatively little behavioural flexibility compared to reactive individuals, in which behaviour patterns appear to be more flexible, making them more responsive to environmental stimuli [26], [42]. Having defined individuals’ behavioural types according to their position on the proactive-reactive axis, our analysis focuses on how their behavioural type relates to individual performance (i.e. maternal mass loss and offspring mass gain). If phenotype-environment mismatch is the mechanism maintaining variation in the proactive/reactive behaviour observed among grey seals, then we hypothesise that variation in individual performance should be relatively high among reactive females. On the other hand, if proactive individuals have evolved a behavioural response that generally performs well in common situations, then variation among their measures of performance may be relatively low. Previous studies have shown that a number of individual and environmental covariates may correlate with maternal behaviour, for example, mother’s prior experience, degree of inter-annual site fidelity, physical and social characteristics of their pupping habitat, pup sex or pup activity [32]–[35]. In order to determine if individual performance is likely to be an indirect result of their proactive-reactive tendencies we test for associations between behavioural type and the above-mentioned covariates. In this case, insignificant associations between behavioural type and the covariate would support the behavioural type as having a direct effect on individual performance.

## Materials and Methods

### Ethics Statement

Grey seals in the UK are currently protected under the Conservation of Seals Act 1970 and the Marine (Scotland) Act 2010. They also fall under the Animals [Scientific Procedures] Act, 1986. All animal handling for this study was approved by and conducted under an UK Home Office license (license number PPL 60/3303) by experienced personnel. The observational and behavioural testing protocols fall out with UK Home office licensed work, but were subjected to ethical review, and approved, by Durham University’s Life Sciences Ethical Review Process (Durham University’s ethics committee). All protocols were designed to conform to the ASAB/ABS Guidelines for the treatment of animals in teaching and research.

### Data Collection

Data were collected during the 2009 (29/9/09 to 31/10/09) and 2010 (29/9/10 to 1/11/10) breeding seasons at the North Rona breeding colony. At North Rona, individual females spend 18–20 days ashore, during which they each bear and nurse one pup [37]. Females generally remain close to their pups throughout lactation, but may occasionally commute between their pup and pools of water [33], [35]. The breeding season lasts 6–8 weeks, thus, there is a turnover of females. Females show a high degree of inter-annual site fidelity (median inter-annual distance moved from previous pupping sites = 55 m [32]), but not all females return to breed every year [32], [37]. Earlier studies used artificial marks to identify individual females at the North Rona colony, but since 1996 mothers’ unique pelage patterns have been catalogued in a photo-ID database to allow recognition without handling [32], [33], [43].

Behavioural consistency in pup-checking rates was assessed in two situations; disturbed and undisturbed. The disturbed situation was generated by the use of an in-field experimental test described in detail in Twiss et al. [31]. In brief, the protocol involves maneuvering a remotely controlled vehicle (RCV) to within 2 m of the focal seal. After a 5 minute period of acclimation an auditory stimulus was played 3 times, each separated by 2 minutes, with the RCV remaining in position for 2 minutes after the last iteration of the stimulus. The auditory stimulus used was a ‘wolf’ call [31], which was chosen as it represented a mildly alarming but natural sound that would also be novel to grey seals on North Rona. Each test was recorded using a digital video camera stationed with the operator located at least 50 m downwind from the target seal. The RCV was stationary within close proximity to the target seal for a total of 11 minutes per test. During this 11 minute period the behavioural metric extracted from the video footage was the focal seal’s pup-checking rate. Focal females were widely spaced around the North Rona study area, with all females tested being geographically separated by at least 20 m [31]. Also, females targeted for the RCV test on a specific day were selected such that no other focal females were exposed to the test on the same day in order to minimise the chance of prior exposure and habituation or sensitisation. RCV tests were repeated on focal seals early and late in lactation, with intervals between tests ranging from 4 to 14 days, however, inter-test interval had no effect upon individual pup-checking rates or degree of variation in individual responses across the two tests [31]. RCV tests were performed both in the 2009 [31] and 2010 breeding seasons (2009∶26 females tested at least once, 19 females tested twice, 2010∶27 females tested at least once, 17 females tested twice; table 1). Seven females were tested in both seasons for both the early and late lactation tests, providing data for an examination of inter-annual consistency of individuals’ mean within-season pup-checking rates (table 1).

**Table 1:** Summary of the number of individual mothers exposed to the RCV test (disturbed situation) and/or recorded in video focals (undisturbed situation) in 2009, 2010 or in both breeding seasons.

	RCV test		Video focal		
Year	At least one	Two tests	At least one	Two focals	RCV test and video focal
2009	26	19	NA	NA	NA
2010	27	17	17	15 (14*)	14
2009 and 2010	10	7	NA	NA	NA

* One mother was disturbed by conspecifics for more than one third of the video footage for one of her video focals, therefore, only 14 individuals had two usable video focals.

Undisturbed pup-checking rates were determined in the 2010 breeding season only, by the use of 30 minute video focal samples. Video footage collected on two days during a female’s lactation period recorded the mother’s behaviour during ‘quiet’ periods of no disturbance (e.g. no aggressive interactions with neighbours, no disturbance from nearby males) and when the mother and pup were clearly in view of each other. Video footage was gathered for 17 females, of which 15 had two video focals (the remaining two departing the colony before a second video focal could be obtained; table 1). These video focals were examined to extract individual pup-checking rates during these relaxed periods, so that rates could be compared with the pup checking rates extracted from the RCV tests. If the females became disturbed during a focal, pup-checking rates were computed based on the undisturbed portion of the video. If the female was disturbed for more than one third of the video, the data were discarded. One female was disturbed by a male for more than 10 minutes of a video focal, whilst the remaining females all had undisturbed pup-checking rates computed based on at least 20 minutes of video footage. Consequently, fourteen females had measures for pup-checking rates (expressed as number of pup-checks performed per minute) from both relaxed and alarmed contexts (table 1).

The same females that were part of the RCV and video focal studies were also subjects within a long term study of individual reproductive performance [37]. As females fast during the breeding season, relying on stored reserves to provision their pup, they effectively constitute a closed system for accurate monitoring of reproductive expenditure (for full details see [37]). Briefly, these females and their pups were captured twice, typically 11 days apart, during their 18–20 day lactation period to determine a range of phenotypic measures of annual reproductive performance in 2010: (1) Maternal post-partum mass (kg) which represents a standard reference point for mother’s mass and is an index of somatic growth and prior foraging success. (2) Maternal daily mass loss rate (kg/day) during lactation which provides a time averaged index of rate of maternal expenditure. (3) Daily rate of pup mass gain (kg/day) representing time averaged pup growth rate. All mothers used in the analyses presented here successfully raised their pup to weaning and none of the mothers were observed nursing pups other than their own pup.

In addition, locations of all seals were mapped daily by PPP, recording individual seal locations with sub-metre accuracy and their identities based on the Photo-ID catalogue [33], [35]. These maps were digitised into an existing Geographical Information System (GIS) database along with sub-metre resolution physical habitat data [34], [35], [44]. At North Rona access to pools of water on the colony is the primary determinant of habitat quality and pupping site choice [34], [35], [44], and the GIS provides data on individual proximity to pools and local conspecific density (number of adult females within a 10 m radius of each individual) and individual nearest adult female neighbour distances (m). As these data were available for each day during a female’s stay ashore (c. 18–20 days [36], [37] ), we calculated the following parameters from the GIS database for females present in 2010; median distance to pool (m), median distance to nearest neighbour (m), median density of adult females within 10 m radius [34]. Daily distribution maps of females also provided accurate pupping date, and duration of lactation for each female. Female grey seals begin to recruit into the breeding population aged approximately 3–4 years [45], and all females included in these analyses were multiparous adults, and had pupped previously on North Rona. Although actual maternal age was known for only four of the females involved in the analyses presented here (ages: 19, 21, 23 and 28 years), long term records of individual female presence at North Rona since the 1996 breeding season provided a minimum estimate of the number of previous breeding attempts for each individual.

Individual time-activity budgets were derived from instantaneous scan sampling [9], [46] of each female’s behaviour at 5 minute intervals during observation hours (0700 to 1800 h BST) spanning the entire 2010 breeding season, using the following broad behavioural categories: resting, alert, comfort move, locomotion, pup-checking, pup interactions, presenting, nursing, aggressive behaviour (classified separately as aggression towards females and aggression towards males), and out of sight (definitions for these categories are presented in [34]). All scan sample observations were made by SDT. In addition, the same scan sampling protocol applied to pups provided measures of the amount of time that pups were active (i.e. not resting) but not suckling. All mothers and pups included in the analyses were scanned more than 200 times (range: 582–1419 scans per female, 424–1299 scans per pup) to ensure that estimates of the time spent in each behavioural category were representative [9], [46]. Finally, hourly records were made of mothers’ proximity to their pups, assessed visually as the number of adult body lengths (1 body length = approximately 2 m [37]), and median values were computed for each mother.

### Statistical Methods

Our initial analyses focused on quantifying the repeatability [47] of individual pup-checking rates within and across situations, and within and across breeding seasons. We used the Intraclass Correlation Coefficient (ICC) as a measure of repeatability [47]–[49] to determine; (i) consistency of individual pup-checking rates across early and late lactation for the RCV tests within years, (ii) consistency of individual mothers’ mean pup-checking rates from early and late lactation for the RCV tests across years, (iii) individual consistency in pup-checking rates between successive video focals for the undisturbed situation in 2010, and (iv) consistency in individual mothers’ mean pup-checking rates in response to the RCV tests in 2010 and their mean undisturbed rates derived from the video focals. Repeatability is a measure of the “degree to which variation within individuals contributes to total variation in a population” [50] and ICC is a widely used measure of the consistency of a particular behaviour through time [47]. We used ICC2 in the *R* [51] package *psych* [52] which is a two-way random effects model considering both individual and sampling intervals as random effects (Case 2 in Shrout and Fleiss [53])**.** For all ICCs shown the number of observations per individual (n_0_) is two [50].

We calculated individual changes in rates of pup-checking from the undisturbed to the disturbed situation in 2010 (mean rate in response to the RCV test – mean rate derived from undisturbed video focals) as a measure of behavioural plasticity across situations, ranging from more proactive (less plastic) to more reactive (more plastic). We used this to define individuals’ behavioural type, according to their position along this proactive-reactive axis.

Our main analysis utilised likelihood ratio tests (LRTs, [54]) to investigate the relationship between behavioural type and maternal reproductive expenditure (daily mass loss rate) and fitness outcome (as measured by pup growth rate). Specifically, we were interested in whether expected expenditure and pup growth and the uncertainty (variation) in these parameters were both related to changes in the mother’s pup-checking rates (behavioural type), and if any detected effects varied with the sex of the pup. We assumed that mean expenditure and pup growth rate were linearly related to the mother’s behavioural type and variation about the mean was normally-distributed with a standard deviation that was linearly related to behavioural type. Tests for changes in the mean and standard deviation of expenditure and pup growth with respect to behavioural type were performed by comparing model fits with and without the corresponding linear term. Evidence for pup sex effects were investigated by comparing the best fitting model that ignored the sex of the offspring with the model that fitted the male and female growth data separately.

To examine whether variation in maternal expenditure and fitness outcome was likely a direct result of behavioural type we tested if behavioural type was associated with other covariates known to be influential in maternal behaviour patterns [32]–[35]. Specifically, we examined potential relations between behavioural type and GIS derived measures of the mothers’ local physical or social environment, and the general behaviour patterns of the mother or their pup derived from the time-activity budget data. In each case we performed a linear regression analysis that incorporated a randomisation test. Randomisation was necessary to account for the non-normal distribution of residuals about the fitted regression line [55]. Our test statistic was the absolute value of the slope of the fitted line (*B*), and we performed 2000 randomisations of each analysis to calculate levels of significance (*p*). We also calculated effect sizes (*b,* [56]), defined as the relative change in the covariate of interest for each standard deviation change in the behavioural type observed among mothers sampled.

## Results

**Figure 1:**
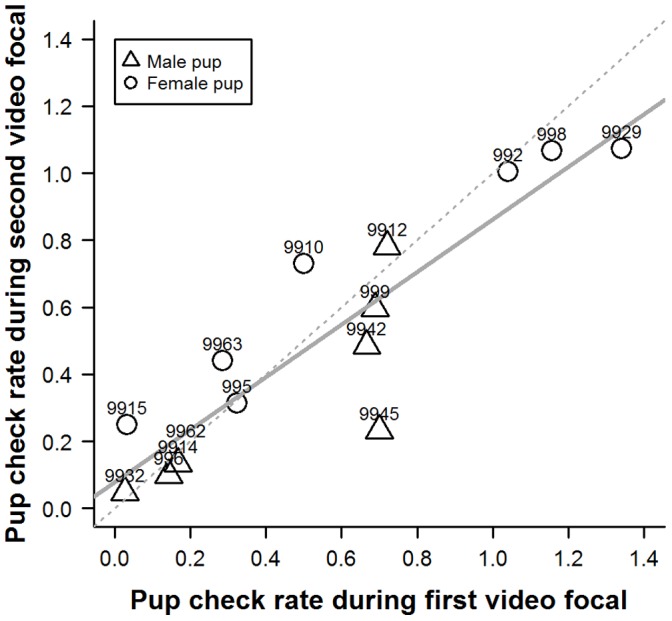
The high degree of repeatability of individuals’ undisturbed pup-checking rates (min^−1^) across the two time points in the 2010 breeding season. Numbers denote individual identities, solid line is line of best fit and dashed line is 1∶1 line.

**Figure 2:**
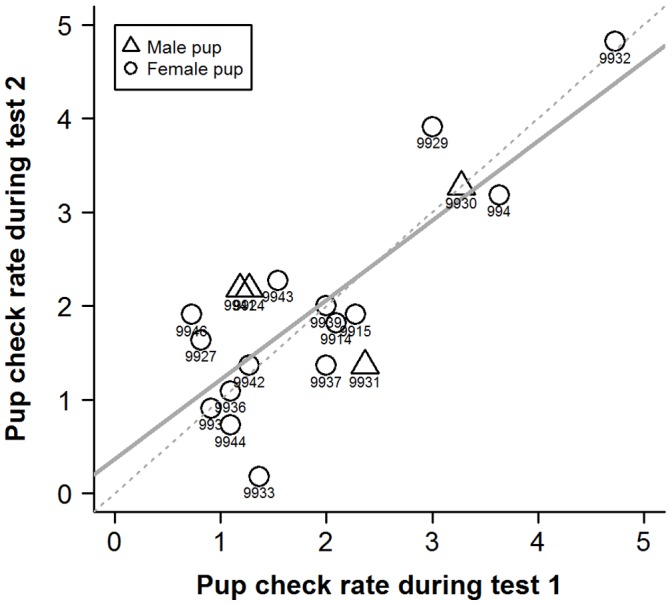
The high degree of repeatability of individuals’ pup-checking rates (min^−1^) in response to the RCV test across the two time points in the 2009 breeding season. Test 1 was conducted early in lactation, test 2 late in lactation. Numbers denote individual identities, solid line is line of best fit and dashed line is 1∶1 line. Adapted from Twiss et al. [31].

**Figure 3:**
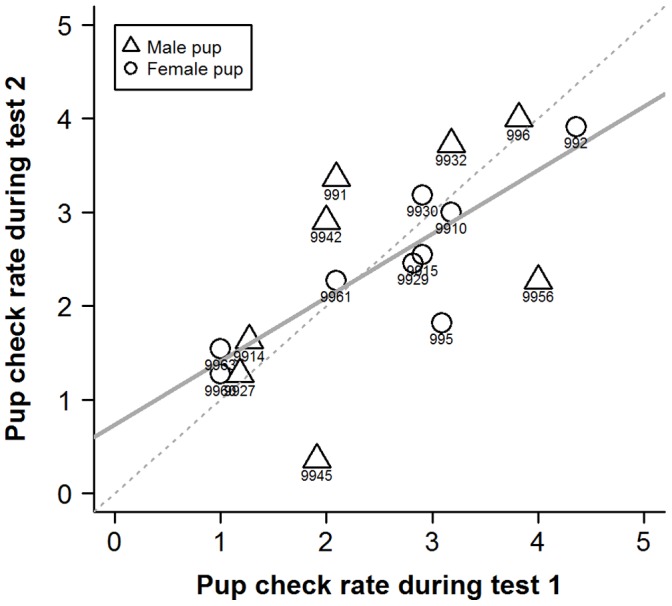
The high degree of repeatability of individuals’ pup-checking rates (min^−1^) in response to the RCV test across the two time points in the 2010 breeding season. Test 1 was conducted early in lactation, test 2 late in lactation. Numbers denote individual identities, solid line is line of best fit and dashed line is 1∶1 line.

**Figure 4:**
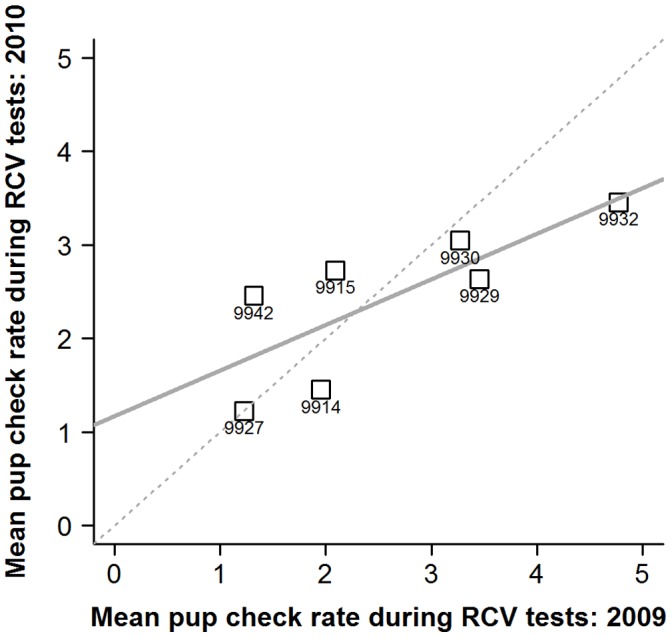
The repeatability of mean individual pup-checking rates (min^−1^) in response to the RCV tests across the two successive breeding seasons. Numbers denote individual identities, solid line is line of best fit and dashed line is 1∶1 line.

**Figure 5:**
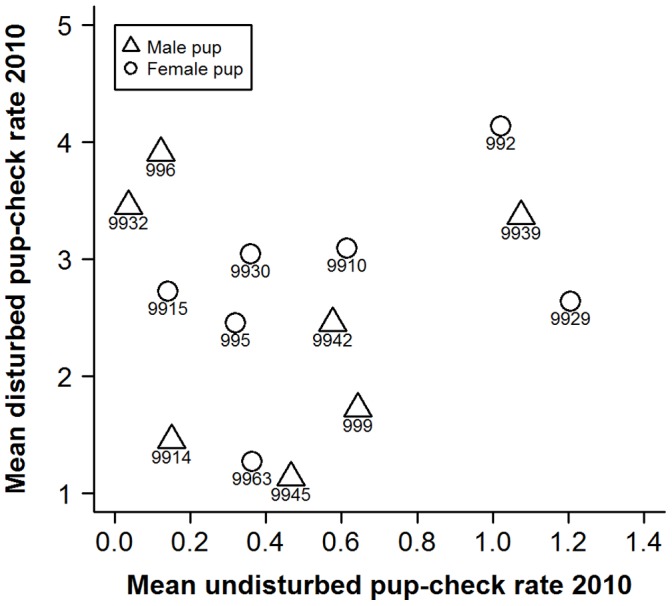
Scatterplot showing the lack of correlation between individuals’ mean undisturbed pup-checking rates and their mean disturbed pup-checking rates in the 2010 season. Numbers represent individual mother identities.

Individual pup-checking rates in the undisturbed context exhibited a high degree of repeatability (figure 1; ICC2 = 0.90, F_14,14_ = 18, p<0.001, 95%CI: 0.72−0.96). Females also showed significant repeatability in their responses to the RCV test in both 2009 (figure 2; ICC2 = 0.81, F_18,18_ = 9.4, p<0.001, 95%CI: 0.58−0.92) and 2010 (figure 3; ICC2 = 0.70, F_16,16_ = 5.5, p<0.001, 95%CI: 0.34−0.88). Across years, individual female’s mean responses over the early and late lactation tests did show a degree of repeatability (figure 4; ICC2 = 0.72, F_6,6_ = 5.6, p = 0.027, 95%CI: 0.011−0.95) although the confidence intervals are noticeably wider than the within year comparisons. However, there was no consistency between individuals’ mean undisturbed pup-checking rates and their mean disturbed pup-checking rates in the 2010 season (figure 5; ICC2 = 0.024, F_13,13_ = 1.28, p = 0.33, 95%CI: −0.055−0.21). When examining how females altered their pup-checking rates between the undisturbed and the disturbed situations we found a range of increases in pup-checking rate from 0.67 min^−1^ to 3.79 min^−1^. We considered females who showed little change in pup-checking rates as more proactive (individuals who maintain a similar level of pup-checking behaviour irrespective of situation), whilst those who showed larger increases in pup-checking rates were considered to be more reactive (individuals who show marked changes in pup-checking rates across situations).

**Figure 6:**
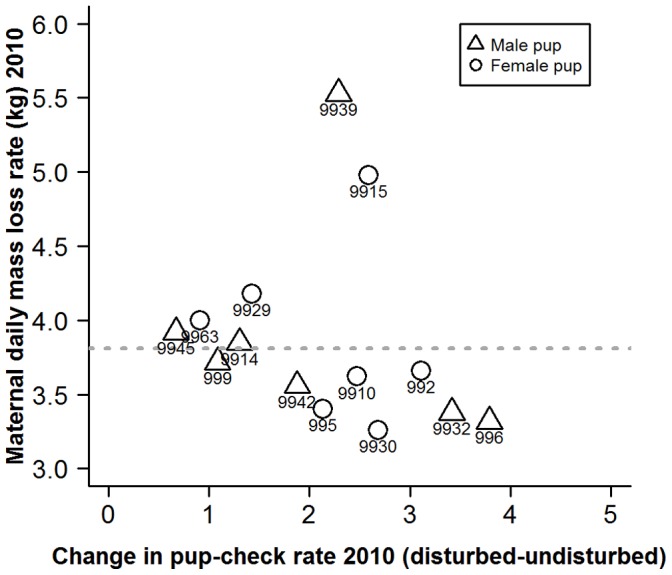
Scatterplot of maternal daily mass loss rates and the degree of change in mother’s pup-checking rate (min^−1^) from undisturbed to disturbed situations. Numbers represent individual mother identities. Horizontal dashed line represents long term population mean maternal daily mass loss rate (3.81±0.055 kg/day) derived from a larger sample (114) of mothers collected over multiple years (2005–2009).

**Figure 7:**
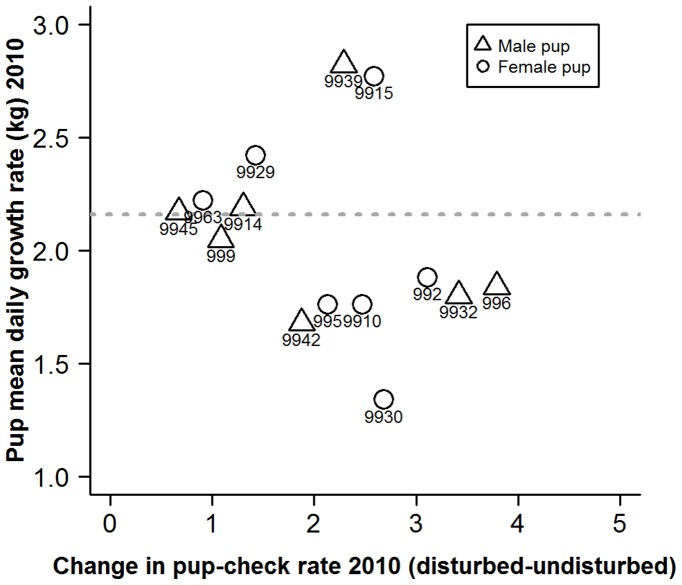
Scatterplot of pup daily growth rates and the degree of change in mother’s pup-checking rate (min^−1^) from undisturbed to disturbed situations. Numbers represent individual mother identities. Horizontal dashed line represents long term population mean pup growth rate (2.16±0.039 kg/day) derived from a larger sample (113) of pups collected over multiple years (2005–2009).

We found evidence that variation in maternal daily mass loss rate was positively associated with behavioural type (LRT; *G*_1_ = 5.75, *p* = 0.016); however, there was no evidence of an association with the mean (LRT; *G*_1_ = 0.02, *p* = 0.892). Also, there was no evidence that offspring sex influenced the pattern of expenditure (LRT; *G*_3_ = 0.66, *p* = 0.882). Thus, although average mass loss was uncorrelated with behavioural type, proactive mothers exhibited similar rates of mass loss; whereas, reactive mothers varied markedly in their rates of mass loss (figure 6). These rates of loss were consistent with those observed between 2005 and 2009 (figure 6). Not surprisingly, as offspring mass gain is highly, positively correlated with maternal mass loss (Pearson’s *r* = 0.93, n = 14, p<0.0001), similar patterns were observed when comparing behavioural type with pup growth rate (figure 7). Specifically, variation in pup growth rate was positively correlated with behavioural type (LRT; *G*_1_ = 5.01, *p* = 0.025), but there was no evidence of an association between behavioural type and mean pup growth rate (LRT, *G*_1_ = 1.07, *p* = 0.302). This pattern was the same for male and female pups (LRT, *G*_3_ = 0.29, *p* = 0.962). Rates of pup mass gain were also similar to those observed between 2005 and 2009 (figure 7).

**Figure 8:**
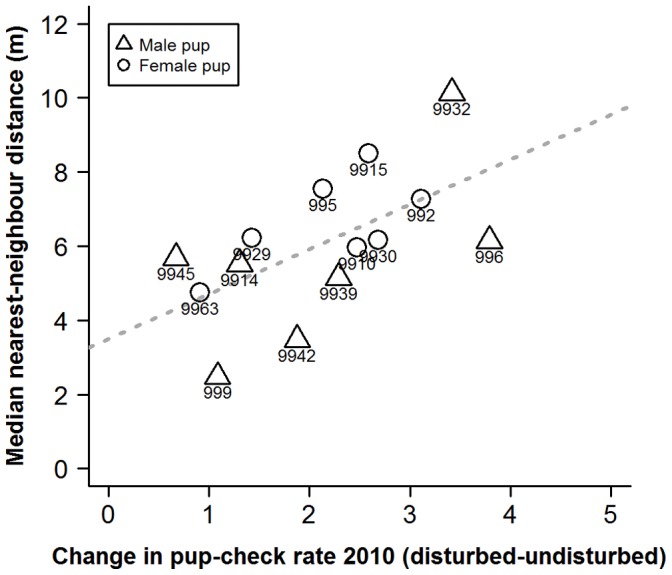
Scatterplot of median nearest neighbour distances (m) and the degree of change in mother’s pup-checking rate (min^−1^) from undisturbed to disturbed situations. Numbers represent individual mother identities. Dashed line represents line of best fit.

There was no significant relationship between a mother’s behavioural type and their median proximity to their own pup or their median proximity to pools of water (table 2). However, more proactive mothers tended to be closer to their nearest female neighbour than more reactive mothers (*p* = 0.04, figure 8, table 2). This was reflected in terms of local conspecific densities, with the more proactive mothers in locations with higher densities than reactive mothers (*p* = 0.05, table 2). However, it should be noted that median numbers of conspecific females within 10 m radii of the study individuals only ranged from 0 to 2 for all but one mother, who had a median value of 5 females within a 10 m radius and a small change in pup-checking rates across situations (1.08 min^−1^). It should also be noted that these relationships are non-significant upon application of a Bonferroni adjustment (table 2). There was no relationship between behavioural type and pupping date, duration of lactation, the extent of pupping site fidelity, the number of years each mother was present between 1996 and 2010, or with the year in which they were first sighted (table 2).

**Table 2:** Results of randomisation tests examining the significance of relationships between females’ positions on the proactive-reactive continuum (as defined by change in pup-checking rate from relaxed to alarmed contexts) and measures of habitat use and colony attendance.

parameter	mean	SE	B	p	b
Median distance to pools of water during lactation (m)	2.58	0.44	−0.06	0.90	−0.022
Median distance to nearest neighbour during lactation (m)	6.07	0.52	1.21	**0.04**	0.191
Median conspecific female density within 10 m radius	1.75	0.30	−0.70	**0.05**	−0.384
Median mother-pup distance (adult body lengths ≈ 2 m)	1.37	0.10	−0.19	0.08	−0.133
Pupping date (days from 1^st^ September)	38.86	1.46	−2.66	0.11	−0.066
Duration of lactation (days)	19.64	0.92	0.64	0.52	0.031
Site fidelity (distance (m) between 2009 and 2010 pupping sites)	44.27	6.55	−11.90	0.13	−0.258
Number of years present between 1996 and 2010	5.14	0.62	0.28	0.68	0.052
Year in which the mother was first seen with a pup	2004	0.93	−0.87	0.41	−0.001

The *p* value is computed using an approach that combines linear regression with a randomisation test. Sample size = 14, with the exception of site fidelity measures where n = 11. Significant results (*p*≤0.05) are in bold, though application of Bonferroni adjustment renders all tests non-significant at p≤0.0063. ***B*** represents slope of the relationship, and ***b*** represents effect size.

**Figure 9:**
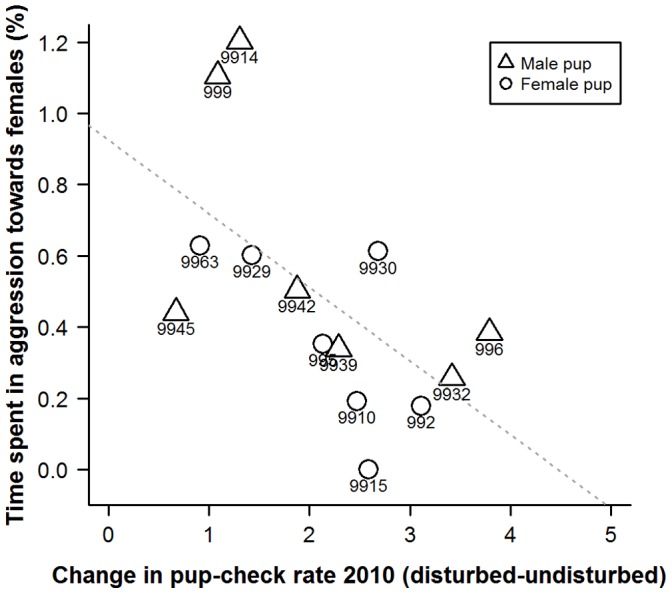
Scatterplot of percentage of time that mothers spent in aggression towards other females and the degree of change in mother’s pup-checking rate (min^−1^) from undisturbed to disturbed situations. Numbers represent individual mother identities. Dashed line represents line of best fit.

There was no overall difference in our metric of behavioural type between mothers with male pups and mothers with female pups (*W* = 21, n_males_ = 7, n_females_ = 7, *p* = 0.71) and there was no relationship between behavioural type and the activity levels of pups (table 3). There was no relationship between behavioural type and the percentage of time that mothers spent in resting, alert, comfort move, locomotion, pup-checking, pup interactions, nursing or presenting (table 3). There was also no relationship in terms of overall percentage of time spent in aggressive behaviour, however, more proactive mothers did spend significantly more time in aggressive activities directed towards other females (*p* = 0.05, figure 9, table 3), although this is potentially an effect of proactive mothers being located closer to neighbouring females (randomisation result of median nearest neighbour distance against aggression towards females: *p* = 0.05, *B* = −0.11, *b* = −0.43). Finally, there was a non-significant trend for more reactive females to exhibit more aggression towards males (*p* = 0.07, table 3). Again, these trends are non-significant if a Bonferroni adjustment is applied.

**Table 3:** Results of randomisation tests examining the significance of relationships between females’ positions on the proactive-reactive continuum (as defined by change in pup-checking rate from relaxed to alarmed contexts) and percentage time spent in various behaviours as determined from time-activity budgets.

Behaviour	mean	SE	B	p	b
Rest	79.09	0.99	−0.48	0.67	−0.006
Alert	6.54	0.51	0.24	0.67	0.035
Comfort Move	2.49	0.17	0.20	0.28	0.077
Locomotion	1.43	0.13	−0.12	0.39	−0.081
Pup-check	2.46	0.19	0.16	0.46	0.063
Pup interaction	1.71	0.29	0.23	0.47	0.129
Nursing	3.95	0.29	−0.16	0.73	−0.039
Presenting	6.20	0.35	−0.03	0.83	−0.005
Total aggression	0.74	0.09	−0.09	0.36	−0.117
Aggression towards females	0.49	0.09	−0.21	**0.05**	−0.412
Aggression towards males	0.25	0.06	0.12	0.07	0.461
Pup activity	19.51	0.90	−0.51	0.59	−0.025

The *p* value is computed using an approach that combines linear regression with a randomisation test. Sample size = 14. Significant results (*p*≤0.05) are in bold, though application of Bonferroni adjustment renders all tests non-significant at p≤0.0042. ***B*** represents slope of the relationship, and ***b*** represents effect size.

## Discussion

Although CIDs in behaviour do not preclude plasticity, they do place limits on the degree of individual plasticity [2], [26], [40], [41], [57]. However, relatively few studies have integrated examination of individual behavioural consistency (a key element of personality) and plasticity, particularly in the wild [40], [41], [57]–[59], although the two are inextricably linked. Here we have shown that female grey seals exhibit behavioural consistency within, but not across situations, and that the degree of behavioural plasticity shown has links to patterns of short term (within season) reproductive performance, with more behaviourally flexible (reactive) mothers exhibiting more variation in reproductive expenditure and consequent pup growth rates.

Female grey seals clearly demonstrate strong individual consistency in their tendency to perform pup-checks in either undisturbed or simulated disturbed situations. These are very high and robust levels of repeatability (0.7 to 0.9) with the lower 95% confidence intervals well above zero. These values lie within the upper range of repeatability estimates presented in a recent meta-analysis of repeatability of animal behaviour, where repeatability measures for field based studies of vertebrates ranged from 0.01 to 0.93 [47]. Although the responses in the disturbed situation across years revealed reasonably high estimates of repeatability, there were wide confidence intervals associated with these measures, with lower confidence limits close to zero. Whilst our smaller sample size for this test may explain this result, it could also be a real effect, and meta-analyses suggest that repeatability tends to decline with time between sampling intervals [47]. It is worth noting here that these female grey seals are exhibiting inter-annual consistency in their pup-checking behaviour despite the fact that their pups’ identity and, in some cases, sex differ across those years. The temporal scale over which CIDs persist and whether behavioural types or personalities are open to gradual modification or age effects remains a largely unexplored area within the field of personality studies [22]. Rarely have laboratory or field studies been able to explore the long term patterns in consistency in long-lived animals, though a few studies of marine mammals have shown long term trends in movement behaviours, for example consistency in West Indian manatee (*Trichechus manatus*) seasonal movement patterns over periods of up to 10 years [60]. Here, we show consistency both within and between consecutive years in a species where females can live to 30–40 years of age [37], [45].

The lack of correlation of individual responses in undisturbed and disturbed situations suggests that individual expressions of pup-checking behaviour are situation specific. In the terms defined by Stamps and Groothius [40], [41], post-partum female grey seals show a high degree of differential consistency, but also exhibit considerable contextual plasticity (noting that Stamps and Groothius’ definition of context “encompasses both ‘situations’ (different ecological conditions) and ‘contexts’ (different functional behavioural categories)” [40]). However, the degree of change in pup-checking rates across these situations suggests a range of behavioural types spanning a proactive-reactive axis [26], [42]. Proactive females tended to maintain a similar level of pup-checking behaviour irrespective of situation, presenting a fairly fixed response to the changing circumstances indicating very limited plasticity [26], [42], [58]. Reactive females however, altered their pup-checking rates markedly across situations, showing a much higher degree of behavioural plasticity and ability to react to the environmental stimuli. Although behavioural consistency is a key component of studies of non-human animal personality, the degree of behavioural plasticity exhibited by an individual may also be a major element of personality [57]–[59]. The extent to which an individual tends to have fixed or canalised responses, or whether they show flexibility in their behavioural responses to environmental cues suggests fundamental differences in the way that resources are allocated to their neurobiological and physiological development during ontogeny [22], [26] perhaps reflecting differing life history strategies [15], [61].

In order to provide insights into whether habitat influenced individual plasticity in pup-checking behaviour we examined spatial and temporal aspects of females’ locations on the colony with respect to their behavioural type. The behavioural type of the mothers studied here did not exhibit any patterns in relation to pupping date, where they were located with respect to key habitat features (i.e. pools [34], [35]) or proximity to their pup. However, the more proactive females tended to be located closer to neighbours and in higher density areas. Although this relationship was non-significant upon Bonferroni adjustment, there was a moderate effect size (19% for neighbour proximity). Females in closer proximity to neighbours and in higher density areas are likely to be exposed to more interactions with conspecifics, and this greater disturbance might be expected to lead to higher pup-checking rates. However, this was not the case for these proactive females which seemed to exhibit a more *laissez-faire* mothering style [62], [63], showing limited change in pup-checking rates, even though they tended to be closer to neighbouring females. One possible explanation is that proactive females habituate to the potentially higher levels of conspecific activity in their higher density locations on the colony [4]. The fact that the individual pup-checking rates in both the undisturbed and disturbed situations showed not only repeatability but also a high level of absolute agreement of values across measurement points (i.e. the line of best fit was close to the 1∶1 line; figures 1, 2, 3) suggests that there was little or no habituation within seasons. However, it might be argued that proactive females, over a number of previous breeding seasons, have habituated to disturbance from conspecifics. In consideration of this, it is interesting to note that among the more proactive mothers (IDs 9945, 9963, 999, 9914, 9929; figures 6 and 7), undisturbed pup-checking rates spanned the entire range observed for all study females (figure 1). Thus, although proactive females are similar in that they show little increase in pup-checking in response to disturbance, they do differ considerably in their ‘baseline’ levels of pup-checking. Whether habituation plays a role in the lack of response to disturbance warrants further study, and could be achieved by using repeated testing with the RCV. Of course, the rate and extent of habituation expressed by individuals may also be an element of personality, but in the framework of the proactive-reactive axis, given that reactive individuals are those that express behavioural flexibility [26], [42], one might expect reactive individuals to habituate more rapidly. Although there has been relatively little research on the links between personality and habituation (or sensitisation or acclimation), studies of various bird species suggest that more aggressive (arguably proactive) individuals take longer to habituate to repeated stimuli than calmer (arguably reactive) individuals (male ring doves, *Streptopelia risoria* [64], great tits, *Parus major* [65], yellow-eyed penguins, *Megadyptes antipodes* [66]), whilst one of the few studies of mammals in this respect found no inter-individual variation in habituation (eastern chipmunks, *Tamias striatus* [4]).

A further potential explanation of the reduced plasticity of proactive females may be a ‘selfish herd’ effect. If proactive mothers are located in areas of higher density they may not need to respond to disturbances so frequently. However, the RCV was a novel stimulus upon first presentation to all females in this study. Also, the RCV was positioned 2 m from the target mother, and in all cases was closer to her than any neighbouring female was to the target seal. All mothers did respond to the RCV by approaching it and placing themselves between the RCV and their pup, suggesting that they did perceive the RCV as a potential threat to their pup [31]. Furthermore, the variation in proactive females’ baseline undisturbed rates of pup-checking would seem to argue against a selfish herd effect. However, the links between neighbourhood density, neighbour activity and behavioural type clearly warrant further study.

There was no indication that proactive and reactive females differed in their prior breeding experience, and all females had successfully weaned pups in previous years. Behavioural type was not related to pup sex and there was no evidence that the more reactive females had pups that were more active than those of more proactive mothers. Therefore, differences in maternal pup-checking behaviour do not seem to be related to differences in pup behaviour or pup sex. A mother’s position on the proactive-reactive axis showed no relationships with components of their time-activity budgets, with the possible exception of patterns of aggression. There was a suggested trend for more proactive mothers to spend more time in aggression with other adult females. Although non-significant upon Bonferroni adjustment, this relationship exhibited a reasonably large effect size (41%). This trend may simply be a product of the proximity of proactive mothers to their neighbours, leading to more aggressive interactions. However, cause and effect is difficult to tease apart here; if proactive females were indeed innately more aggressive [26], [42], this might enable them to access and monopolise the higher density areas on the colony which occur around preferred and advantageous habitat (i.e. locations that provide good access to pools of water [32]–[35]). Laboratory studies of rats and mice have shown greater aggressiveness in proactive individuals [26], [42].

There was a non-significant trend towards the more reactive mothers spending more time in aggression with males, but again with a large effect size (46%). This may be a product of their tendency to be found in lower density areas subject to more transient male incursions and, therefore, male harassment [67]. Curiously, there was no indication that the more reactive mothers spent significantly more time engaged in pup-checking behaviour (as determined by the scan sampling protocol). It might be expected that reactive mothers would exhibit more time spent pup-checking due to their elevated pup-checking responses to disturbances. However, a mother’s position on the proactive-reactive axis depends solely on the degree of change in pup-checking from undisturbed to disturbed situations, not on the absolute baseline (undisturbed) levels, and some proactive mothers expressed relatively high levels of pup-checking irrespective of situation. Also, if the more reactive mothers select, or are limited to, pupping locations further from other mothers, they may experience lower disturbance overall, at least until males become attentive towards the end of lactation.

A major area of debate in the field of personality research is how CIDs in behaviour are maintained in the face of selection, and whilst many theoretical explanations have been proposed [1], [16] there are few field-based empirical insights into potential mechanisms [20], [28], [30], [68], [69]. Our models of variation in maternal expenditure and pup growth rates with respect to pup-checking plasticity confirmed that with increasing plasticity in pup-checking across situations, mean expenditure and pup growth rates remained constant, but variation increases. These findings suggest no overall difference between proactive and reactive mothers’ average reproductive expenditure and consequent success (assessed by pup growth rate), but greater variation in expenditure and success among the more behaviourally flexible reactive mothers. This presents a possible mechanism that might lead to the stable coexistence of proactive and reactive behavioural types within the population [1], [14], [16], [70]. It remains unclear quite how behavioural flexibility translates into more varied reproductive investment and success. However, spatial and temporal environmental heterogeneity has also been shown to maintain behavioural diversity between individuals [16], [71], [72], which suggests that certain types of individuals may be more successful under different environmental situations than others [20]. Grey seal reproductive success is affected by spatial and temporal variations in fine scale breeding habitat [33]–[35]. It has been argued that proactive individuals may be adapted to stable environmental conditions, whereas reactive individuals may cope better with variable and unpredictable environmental conditions [26]. However, our results suggest that there is a trade-off here, with proactive females adopting a strategy that fits their phenotype reasonably well to the most common environmental conditions, whilst minimising the costs of plasticity [12], [13], but rarely achieving a perfect phenotype-environment match. Consequently, proactive mothers tend to achieve average fitness payoffs. Conversely, reactive females attempt to adjust their phenotype to prevailing conditions, potentially achieving a highly rewarding match of phenotype and environment, but they are also subject to the potential costs of plasticity including imperfect phenotype-environment matching [12], [13], [16], leading to greater variation in fitness payoffs.

Maternal post-partum mass clearly plays a key role in determining levels of maternal expenditure and pup growth rates [37], and the data presented here are no exception. Amongst the more reactive mothers, the two with the highest pup growth rates (figure 7) were those with greatest post-partum mass; 256 kg and 224 kg, compared to other reactive mothers with lower pup growth rates whose post-partum masses ranged from 164 to 208 kg whilst the more proactive mothers ranged from 185 kg to 228 kg. It is unclear whether these mass differences are a result of foraging success in the months prior to the 2010 breeding season, or whether they represent individual differences in developmental trajectories over the individuals’ lifetimes. Either way, these results imply that some reactive females do achieve a good fit of phenotype and environment, either in terms of annual access to resources, or access to resources over their lifetime. There may also be size-independent intrinsic differences in maternal quality, such as production of higher quality milk [73], [74]. Such links between maternal quality and behavioural types warrant further attention.

The determinants of behavioural type remain unresolved. Investigations into genetic differences between behavioural types could provide insights into proximate mechanisms and evolutionary consequences [17], [75], [76], particularly in the proactive-reactive spectrum where putative physiological and neuroendocrine mechanisms have already been identified. It has been suggested that behavioural expression in proactive individuals is linked to increased vasopressinergic activity in brain regions that are linked to stress coping, whilst reactive individuals exhibit increased oxytocinergic activity in the same brain regions [26], making candidate gene approaches potentially tractable [76]. However, it is equally likely that developmental processes may have a considerable influence on an individuals’ behavioural type, either through parental effects [77], early experience [22], [61], [78], [79] or links with condition and physiology [15]. A particularly intriguing question is how these proactive and reactive female grey seals behave at sea. Grey seals temporally separate foraging and breeding, and individuals show great variation in location and use of habitat at sea [80]–[82]. The variation in behavioural plasticity shown here raises enticing parallels to the concepts of specialist and generalist foragers [83]–[88] and if grey seal females show similar degrees of low (proactive) and high (reactive) plasticity in behaviours associated with foraging there could be important implications for how the behavioural types fare in changing environments [4], [20], [89], [90] and for ecosystem models [91], [92].

## References

[1] Dall SRX, Houston AI, McNamara JM (2004) The behavioural ecology of personality: consistent individual differences from an adaptive perspective. Ecology Letters 7: 734–739.

[2] Sih A, Bell A, Johnson JC (2004) Behavioural syndromes: an ecological and evolutionary overview. Trends in Ecology and Evolution 19: 372–377. 16701288 10.1016/j.tree.2004.04.009

[3] Bell AM (2007) Future directions in behavioural syndromes research. Proceedings of the Royal Society of London B 274: 755–761. 10.1098/rspb.2006.0199 PMC1919401 17251088

[4] Martin JGA, Réale D (2008) Temperament, risk assessment and habituation to novelty in eastern chipmunks, *Tamias striatus* . Animal Behaviour 75: 309–318.

[5] Sih A, Bell AM (2008) Insights for behavioral ecology from behavioral syndromes. Advances in the Study of Behavior 38: 227–281. 24991063 10.1016/S0065-3454(08)00005-3 PMC4075144

[6] Briffa M, Weiss A (2010) Animal Personality. Current Biology 20: R912–R914. 21056827 10.1016/j.cub.2010.09.019

[7] Briffa M, Greenaway J (2011) High *in situ* repeatability of behaviour indicates animal personality in the Beadlet Anemone *Actinia equina* (Cnidaria). PLoS ONE 6(7): e21963 DOI:10.1371/journal.pone.0021963. 21755015 10.1371/journal.pone.0021963 PMC3130786

[8] Réale D, Gallant BY, Leblanc M, Festa-Bianchet M (2000) Consistency of temperament in bighorn ewes and correlates with behaviour and life history. Animal Behaviour 60: 589–597. 11082229 10.1006/anbe.2000.1530

[9] Twiss SD, Franklin J (2010) Individually consistent behavioural patterns in wild, breeding male grey seals (*Halichoerus grypus*). Aquatic Mammals 36: 234–238.

[10] Dingemanse NJ, Réale D (2005) Natural selection and animal personality. Behaviour 142: 1159–1184.

[11] Réale D, Reader SM, Sol D, McDougall PT, Dingemanse NJ (2007) Integrating animal temperament within ecology and evolution. Biological Reviews 82: 291–318. 17437562 10.1111/j.1469-185X.2007.00010.x

[12] DeWitt TJ, Sih A, Wilson DS (1998) Costs and limits of phenotypic plasticity. Trends in Ecology and Evolution 13: 77–81. 21238209 10.1016/s0169-5347(97)01274-3

[13] Duckworth R (2010) Evolution of personality: Developmental constraints on behavioral flexibility. The Auk 127: 752–758.

[14] Wolf M, van Doorn GS, Leimar O, Weissing FJ (2007) Life-history trade-offs favour the evolution of animal personalities. Nature 447: 581–584. 17538618 10.1038/nature05835

[15] Biro PA, Stamps JA (2010) Do consistent individual differences in metabolic rate promote consistent individual differences in behaviour? Trends in Ecology and Evolution 25: 653–659. 20832898 10.1016/j.tree.2010.08.003

[16] Wolf M, Weissing FJ (2010) An explanatory framework for adaptive personality differences. Philosophical Transactions of the Royal Society B 365: 3959–3968 DOI:10.1098/rstb.2010.0215. 10.1098/rstb.2010.0215 PMC2992748 21078648

[17] Bell AM, Aubin-Horth N (2010) What can whole genome expression data tell us about the ecology and evolution of personality? Philosophical Transactions of the Royal Society B 365: 4001–4012. 10.1098/rstb.2010.0185 PMC2992745 21078652

[18] Stamps JA (2007) Growth-mortality tradeoffs and “personality traits” in animals. Ecology Letters 10: 355–363. 17498134 10.1111/j.1461-0248.2007.01034.x

[19] Biro PA, Stamps JA (2008) Are animal personality traits linked to life-history productivity. Trends in Ecology and Evolution 23: 361–368. 18501468 10.1016/j.tree.2008.04.003

[20] Dingemanse NJ, Both C, Drent PJ, Tinbergen JM (2004) Fitness consequences of avian personalities in a fluctuating environment. Proceedings of the Royal Society of London B 271: 847–852 DOI:10.1098/rspb.2004.2680. 10.1098/rspb.2004.2680 PMC1691663 15255104

[21] Duckworth RA, Kruuk LEB (2009) Evolution of genetic integration between dispersal and colonization ability in a bird. Evolution 63: 968–977 DOI:10.1111/j. 1558–5646.2009.00625.x. 19154391 10.1111/j.1558-5646.2009.00625.x

[22] Groothuis TGG, Trillmich F (2011) Unfolding personalities: The importance of studying ontogeny. Developmental Psychobiology 53: 641–655. 21866544 10.1002/dev.20574

[23] Wilson DS, Coleman K, Clark AB, Biederman L (1993) Shy-bold continuum in pumpkinseed sunfish (*Lepomis gibbosus*): An ecological study of a psychological trait. Journal of Comparative Psychology 107: 250–260.

[24] Archard GA, Braithwaite VA (2010) The importance of wild populations in studies of animal temperament. Journal of Zoology 281: 149–160.

[25] Herborn KA, Macleod R, Miles WTS, Schofield ANB, Alexander L, et al (2010) Personality in captivity reflects personality in the wild. Animal Behaviour 79: 835–843.

[26] Koolhaas JM, de Boer SF, Coppens CM, Buwalda B (2010) Neuroendocrinology of coping styles: Towards understanding the biology of individual variation. Frontiers in Neuroendocrinology 31: 307–321. 20382177 10.1016/j.yfrne.2010.04.001

[27] Minderman J, Reid JM, Evans PGH, Whittingham MJ (2009) Personality traits in wild starlings: exploration behavior and environmental sensitivity. Behavioral Ecology 20: 830–837.

[28] Réale D, Festa-Bianchet M (2003) Predator-induced natural selection on temperament in bighorn ewes. Animal Behaviour 65: 463–470. 10.1006/anbe.2000.1530 11082229

[29] Smith BR, Blumstein DT (2008) Fitness consequences of personality: a meta-analysis. Behavioral Ecology 19: 448–455.

[30] Couchoux C, Cresswell W (2012) Personality constraints versus flexible antipredation behaviors: How important is boldness in risk management of redshanks (*Tringa totanus*) foraging in a natural system? Behavioral Ecology 23: 290–301 DOI:10.1093/beheco/arr185.

[31] Twiss SD, Culloch R, Pomeroy PP (2011) An in-field experimental test of pinniped behavioral types. Marine Mammal Science. DOI: 10.1111/j.1748–7692.2011.00523.x

[32] Pomeroy PP, Anderson SS, Twiss SD (1994) McConnell (1994) Dispersion and site fidelity of breeding female grey seals (*Halichoerus grypus*) on North Rona, Scotland. Journal of Zoology 233: 429–447.

[33] Redman P, Pomeroy PP, Twiss SD (2001) Grey seal maternal attendance patterns are affected by water availability on North Rona, Scotland. Canadian Journal of Zoology 79: 1073–1079.

[34] Twiss SD, Caudron A, Pomeroy PP, Thomas CJ, Mills JP (2000) Fine scale topographical influences on the breeding behaviour of female grey seals. Animal Behaviour 59: 327–338. 10675255 10.1006/anbe.1999.1320

[35] Twiss SD, Thomas CJ, Poland VF, Graves JA, Pomeroy PP (2007) The impact of climatic variation on the opportunity for sexual selection. Biology Letters 3: 2–15 DOI: 10.1098/rsbl.2006.0559. 10.1098/rsbl.2006.0559 PMC2373809 17443953

[36] Twiss SD, Pomeroy PP, Graves JA, Poland VF (2006) Finding fathers - spatio-temporal analysis of paternity assignment in grey seals (*Halichoerus grypus*). Molecular Ecology 15: 1939–1953. 16689909 10.1111/j.1365-294X.2006.02927.x

[37] Pomeroy PP, Fedak MA, Rothery P, Anderson S (1999) Consequences of maternal size for reproductive expenditure and pupping success of grey seals at North Rona, Scotland. Journal of Animal Ecology 68: 235–253.

[38] Kovacs KM (1987) Maternal behaviour and early behavioural ontogeny of grey seals (*Halichoerus grypus*) on the Isle of May, UK. Journal of Zoology 213: 697–715.

[39] Sih A, Bell A, Johnson JC, Ziemba R (2004) Behavioral syndromes: An integrative overview. Quarterly Review of Biology 79: 241–277. 15529965 10.1086/422893

[40] Stamps J, Groothuis TGG (2010) The development of animal personality: Relevance, concepts and perspectives Biological Reviews. 85: 301–325 DOI:10.1111/j.1469–185X.2009.00103.x. 10.1111/j.1469-185X.2009.00103.x 19961473

[41] Stamps J, Groothuis TGG (2010) Developmental perspectives on personality: Implications for ecological and evolutionary studies of individual differences. Philosophical Transactions of the Royal Society B 365: 4029–4041 DOI:10.1098/rstb.2010.0218. 10.1098/rstb.2010.0218 PMC2992751 21078655

[42] Koolhaas JM, Korte SM, De Boer SF, Van der Vegt BJ, Van Reenen CG, et al (1999) Coping styles in animals: Current status in behavior and stress physiology. Neuroscience and Biobehavioral Reviews 23: 925–935 DOI:10.1016/S0149–7634(99)00026–3. 10580307 10.1016/s0149-7634(99)00026-3

[43] Smout S, King R, Pomeroy P (2011) Estimating demographic parameters for capture–recapture data in the presence of multiple mark types. Environmental and Ecological Statistics 18: 331–347 DOI: 10.1007/s10651–010–0135-y.

[44] Twiss SD, Thomas CJ, Pomeroy PP (2001) Topographic spatial characterisation of grey seal *Halichoerus grypus* breeding habitat at a seal’s perceptual spatial grain. Ecography 24: 257–266.

[45] Hewer H R (1964) The determination of age, sexual maturity, longevity and a life-table in the grey seal (*Halichoerus grypus*). Proceedings of the Royal Society of London B 142: 593–624.

[46] Altmann J (1974) Observational study of behaviour: Sampling methods. Behaviour 49: 227–267. 4597405 10.1163/156853974x00534

[47] Bell A M, Hankison SJ, Laskowski KL (2009) The repeatability of behaviour: A meta-analysis. Animal Behaviour 77: 771–783. 24707058 10.1016/j.anbehav.2008.12.022 PMC3972767

[48] Lessels CM, Boag PT (1987) Unrepeatable repeatabilities: A common mistake. The Auk 104: 116–121.

[49] Hayes JP, Jenkins SH (1997) Individual variation in mammals. Journal of Mammalogy 78: 274–293.

[50] Boake CRB (1989) Repeatability: Its role in evolutionary studies of mating behavior. Evolutionary Ecology 3: 173–182.

[51] R Development Core Team. (2010) R: A language and environment for statistical computing [Internet]. Vienna (Austria): R Foundation for Statistical Computing. ISBN: 3–900051–07–0. Available from: http://www.R-project.org/.

[52] Revelle W (2010) psych: Procedures for Personality and Psychological Research Northwestern University, Evanston, http://personality-project.org/r/psych.manual.pdf, 1.0–93.

[53] Shrout PE, Fleiss JL (1979) Intraclass correlations: Uses in assessing reliability. Psychological Bulletin 86: 420–428. 18839484 10.1037//0033-2909.86.2.420

[54] Sokal RR, Rohlf FJ (1995) Biometry: The principles and practice of statistics in biological research. New York, Freeman.

[55] Roff DA (2006) Introduction to computer-intensive methods of data analysis in biology. Cambridge University Press.

[56] Nakagawa S (2004) A farewell to Bonferroni: the problems of low statistical power and publication bias. Behavioral Ecology 15: 1044–1045.

[57] Dingemanse NJ, Kazem AJN, Réale D, Wright J (2010) Behavioural reaction norms: Animal personality meets individual plasticity. Trends in Ecology and Evolution 25: 81–89 DOI:10.1016/j.tree.2009.07.013. 19748700 10.1016/j.tree.2009.07.013

[58] Coppens CM, de Boer SF, Koolhaas JM (2010) Coping styles and behavioural flexibility: Towards underlying mechanisms. Philosophical Transactions of the Royal Society B 365: 4021–4028 DOI: 10.1098/rstb.2010.0217. 10.1098/rstb.2010.0217 PMC2992750 21078654

[59] Betini GS, Norris DR (2012) The relationship between personality and plasticity in tree swallow aggression and the consequences for reproductive success. Animal Behaviour 83: 137–143.

[60] Deutsch CJ, Reid JP, Bonde RK, Easton DE, Kochman HI, et al (2003) Seasonal movements, migratory behavior, and site fidelity of West Indian Manatees along the Atlantic coast of the United States. Wildlife Monographs 151: 1–77.

[61] Nussey DH, Clutton-Brock TH, Elston DA, Albon SD, Kruuk LEB (2005) Phenotypic plasticity in a maternal trait in red deer. Journal of Animal Ecology 74: 387–396.

[62] Albers PCH, Timmermans PJA, Vossen JMH (1999) Evidence for the existence of mothering styles in Guinea Pigs (*Cavia aperea f. porcellus*). Behaviour 136: 469–479.

[63] Bardi M, Huffman MA (2002) Effects of maternal style on infant behaviour in Japanese Macaques (*Macaca fuscata*). Developmental Psychobiology 48: 1–9. 10.1002/dev.10065 12430160

[64] Vowles DM, Prewitt E (1971) Stimulus and response specificity in the habituation of anti-predator behaviour in the ring dove (*Streptopelia risoria*). Animal Behaviour 19: 80–86. 5170041 10.1016/s0003-3472(71)80138-0

[65] Carere C, Drent PJ, Koolhaas JM, Groothuis TGG (2004) Personalities in great tits, *Parus major*: stability and consistency. Animal Behaviour 70: 795–805.

[66] Ellenberg U, Mattern T, Seddon PJ (2009) Habituation potential of yellow-eyed penguins depends on sex, character and previous experience with humans. Animal Behaviour 77: 289–296.

[67] Boness DJ, James H (1979) Reproductive behaviour of the grey seal (*Halichoerus grypus*) on Sable Island, Nova Scotia. Journal of Zoology 188: 477–500.

[68] Dingemanse NJ, Both C, van Noordwijk AJ, Rutten AL, Drent PJ (2003) Natal dispersal and personalities in great tits (*Parus major*). Proceedings of the Royal Society of London B 270: 741–747 DOI: 10.1098/rspb.2002.2300. 10.1098/rspb.2002.2300 PMC1691302 12713749

[69] Sih A, Kats LB, Maurer EF (2003) Behavioural correlations across situations and the evolution of antipredator behaviour in a sunfish-salamander system. Animal Behaviour 65: 29–44 DOI: 10.1006/anbe.2002.2025.

[70] Carere C, Caramaschi D, Fawcett TW (2010) Covariation between personalities and individual differences in coping with stress: Converging evidence and hypotheses. Current Zoology 56: 728–740.

[71] Kassen R (2002) The experimental evolution of specialists, generalists, and the maintenance of diversity. Journal of Evolutionary Biology 15: 173–190.

[72] Boon AK, Réale D, Boutin S (2007) The interaction between personality, offspring fitness and food abundance in North American red squirrels. Ecology Letters 10: 1094–1104 doi: 10.1111/j.1461-0248.2007.01106.x. 17877738 10.1111/j.1461-0248.2007.01106.x

[73] Mellish JA E, Iverson SJ, Bowen WD (1999) Variation in milk production and lactation performance in grey seals and consequences for pup growth and weaning characteristics. Physiological and Biochemical Zoology 72: 677–690. 10603331 10.1086/316708

[74] Lang SLC, Iverson SJ, Bowen WD (2009) Repeatability in lactation performance and the consequences for maternal reproductive success in gray seals. Ecology 90: 2513–2523. 19769129 10.1890/08-1386.1

[75] Dochtermann NA, Roff DA (2010) Applying a quantitative genetics framework to behavioural syndrome research. Philosophical Transactions of the Royal Society B 365: 4013–4020. 10.1098/rstb.2010.0129 PMC2992739 21078653

[76] van Oers K, Mueller JC (2010) Evolutionary genomics of animal. Philosophical Transactions of the Royal Society B 365: 3991–4000. 10.1098/rstb.2010.0178 PMC2992743 21078651

[77] Reddon AR (2012) Parental effects on animal personality. Behavioral Ecology 23: 242–245.

[78] Carere C, Drent PJ, Koolhaas JM, Groothuis TGG (2005) Epigenetic effects on personality traits: Early food provisioning and sibling competition. Behaviour 142: 1335–1361.

[79] Groothuis TGG, Carere C (2005) Avian personalities: Characterization and epigenesis. Neuroscience and Biobehavioral Reviews 29: 137–150. 15652261 10.1016/j.neubiorev.2004.06.010

[80] McConnell BJ, Fedak MA, Lovell P, Hammond PS (1999) Movements and foraging areas of grey seals in the North Sea. Journal of Applied Ecology 36: 573–590.

[81] Tucker S, Bowen WD, Iverson SJ (2007) Dimensions of diet segregation in grey seals *Halichoerus grypus* revealed through stable isotopes of carbon (δ^13^C) and nitrogen (δ^15^N). Marine Ecology Progress Series 339: 271–282.

[82] Tucker S, Bowen WD, Iverson SJ (2008) Convergence of diet estimates derived from fatty acids and stable isotopes within individual grey seals. Marine Ecology Progress Series 354: 267–276.

[83] Bolnick DI, Svanbäck R, Fordyce JA, Yang LH, Davis JM, et al (2003) The ecology of individuals: incidence and implications of individual specialization. American Naturalist 161: 1–28. 10.1086/343878 12650459

[84] Estes JA, Riedman ML, Staedler MM, Tinker MT, Lyon BE (2003) Individual variation in prey selection by sea otters: patterns, causes and implications. Journal of Animal Ecology 72: 144–155.

[85] Cherel Y, Hobson KA, Guinet C, Vanpe C (2007) Stable isotopes document seasonal changes in trophic niches and winter foraging individual specialization in diving predators from the Southern Ocean. Journal of Animal Ecology 76: 826–836. 17584388 10.1111/j.1365-2656.2007.01238.x

[86] Tinker MT, Costa DP, Estes JA, Wieringa N (2007) Individual dietary specialization and dive behaviour in the California sea otter: Using archival time-depth data to detect alternative foraging strategies. Deep-Sea Research Part II -Topical Studies in Oceanography 54: 330–342.

[87] Cherel Y, Kernaleguen L, Richard P, Guinet C (2009) Whisker isotopic signature depicts migration patterns and multi-year intra- and inter-individual foraging strategies in fur seals. Biology Letters 5: 830–832. 19793740 10.1098/rsbl.2009.0552 PMC2828010

[88] Newsome SD, Tinker MT, Monson DH, Oftedal OT, Ralls K, et al (2009) Using stable isotopes to investigate individual diet specialization in California sea otters (*Enhydra lutris nereis*). Ecology 90: 961–974. 19449691 10.1890/07-1812.1

[89] Brodie ED, Russell NH (1999) The consistency of individual differences in behaviour: temperature effects on antipredator behaviour in garter snakes. Animal Behaviour 57: 445–451. 10049485 10.1006/anbe.1998.0990

[90] Bell AM, Sih A (2007) Exposure to predation generates personality in threespined sticklebacks (*Gasterosteus aculeatus*). Ecology Letters 10: 828–834. 17663716 10.1111/j.1461-0248.2007.01081.x

[91] Gerber LR (2006) Including behavioral data in demographic models improves estimates of population viability. Frontiers in Ecology and the Environment 4: 419–427.

[92] Austin D, Bowen WD, McMillan JI (2004) Intraspecific variation in movement patterns: modeling individual behaviour in a large marine predator. Oikos 105: 15–30.

